# Apathy following Bilateral Deep Brain Stimulation of Subthalamic Nucleus and Globus Pallidus Internus in Parkinson's Disease: A Meta-Analysis

**DOI:** 10.1155/2022/4204564

**Published:** 2022-10-03

**Authors:** Song Zhang, Shumei Zi, Sihuai Xiong, Heng Peng, Kejia Hu, Hua He

**Affiliations:** ^1^Departments of Neurosurgery, The Third Affiliated Hospital, Naval Medical University, Shanghai 200438, China; ^2^College of Ecology, Lanzhou University, Tianshui Sourth Road 222#, Lanzhou 730000, Gansu, China; ^3^Department of Mathematics, Hong Kong Baptist University, The Hong Kong Special Administrative Region, Kowloon Tong, China; ^4^Department of Neurosurgery, Ruijin Hospital, Shanghai Jiao Tong University School of Medicine, No. 197 Second Ruijin Street, Shanghai 200025, China; ^5^Center for Functional Neurosurgery, Ruijin Hospital, Shanghai Jiao Tong University School of Medicine, Shanghai 200025, China

## Abstract

Parkinson's disease (PD) is a progressive neurodegenerative disorder typically manifested by its motor symptoms. In addition, PD patients also suffer from many nonmotor symptoms (NMSs), such as apathy. Bilateral deep brain stimulation (DBS) of the subthalamic nucleus (STN) and the globus pallidus internus (GPi) are recommended as therapeutic interventions for PD, given their pronounced benefit in reducing troublesome dyskinesia. Apathy, a mood disorder recognized as a NMS of PD, has a negative impact on the prognosis of PD patients. However, the effect of STN-DBS and GPi-DBS on apathy is controversial. In the current meta-analysis, we analyzed apathy following bilateral STN-DBS and GPi-DBS in PD patients. Relevant literature was retrieved from public databases, including PubMed, Cochrane Library, and Embase. Studies were included in our analysis based on the following criterion: such studies should report apathy scores presurgery and postsurgery determined by using the Starkstein Apathy Scale or Apathy Evaluation Scale in patients receiving STN or GPi-DBS with at least three months of follow-up. Upon applying this strict criterion, a total of 13 out of 302 studies were included in our study. A mean difference (MD) and 95% confidence interval (CI) were calculated to show the change in apathy scores. We found a statistically significant difference between the presurgery and postsurgery scores in patients receiving STN-DBS (MD = 2.59, 95% CI = 2.23–2.96, *P* < 0.00001), but not in patients receiving GPi-DBS (MD = 0.32, 95% CI = −2.78–3.41, *P*=0.84). STN-DBS may worsen the condition of apathy, which may result from the reduction of dopaminergic medication. In conclusion, STN-DBS seems to relatively worsen the condition of apathy compared to GPi-DBS. Further studies should focus on the mechanisms of postoperatively apathy and the degree of apathy in STN-DBS versus GPi-DBS.

## 1. Introduction

Parkinson's disease (PD) is a progressive neurodegenerative disorder typically manifested by involuntary movements such as tremor, bradykinesia, rigidity, and gait disturbance [1]. In addition to these motor symptoms, PD patients also suffer from several NMS including mood disorders and cognitive changes [2]. For example, depression and apathy are both common NMS. They belong to mood disorders and cognitive changes, respectively. More specifically, depression includes sadness and negative self-thoughts, while apathetic people lack the ability to respond to both negative and positive events [3]. Dopamine medications reduce the motor symptoms effectively. However, long-term treatment may cause side effects such as response fluctuations, dyskinesias, and impulse control disorders [4]. Thus, deep brain stimulation (DBS) of the subthalamic nucleus (STN) and the globus pallidus internus (GPi) are strongly recommended as therapeutic interventions for PD to alleviate motor symptoms [5]. Compared to dopamine treatment, DBS leads to better outcomes in alleviating motor symptoms and improving patients' prognosis [6].

In recent years, there have been many research on the postoperative status of PD patients postDBS. Apathy, a mood disorder, is a gradually recognized NMS of PD, commonly described as the loss of motivation, decreased initiative, interest, and energy, and emotional indifference with flattened affect [7]. Apathy has a negative impact on the long-term prognosis of PD patients and can significantly increase the burden on caregivers [8]. Whether STN-DBS affects apathy in patients is still debated. While some studies suggest that apathy is aggravated in PD patients after STN-DBS [9–14]. Other studies have concluded the opposite [15–17]. Recently, a new meta-analysis showed increased apathy after STN-DBS compared to the preoperative state or a control group only treated with medication, contrary to the nonmotor manifestation of PD [18]. In terms of therapeutic efficacy, the degree of motor symptom reduction was equivalent in GPi-DBS and STN-DBS [19]. However, compared to a large number of studies on the correlation between STN-DBS and apathy, only a few studies has examined the correlation between apathy and GPi-DBS, and there is no meta-analysis associated with it. At the same time, few articles have compared the prognostic differences between STN-DBS and GPi-DBS, especially in terms of apathy, and no studies have been conducted qualitatively and quantitatively to analyze the increase in apathy after stimulation.

Therefore, we performed a meta-analysis to study the effect of bilateral GPi-DBS on apathy compared with the preoperative state and verify increased apathy in PD patients after bilateral STN-DBS by collecting more up-to-date evidence. We hope to qualitatively compare the differences in the effects of GPi-DBS and STN-DBS on apathy by comparing the differences in apathy between presurgeryand postsurgery patients, which will provide a better reference for the choice of DBS in patients with advanced PD, especially those suffering from severe apathy.

## 2. Materials and Methods

### 2.1. Literature Search Strategy

We conducted a systematic search for relevant articles in PubMed, Cochrane Library, and Embase up to January 2022. The following keywords were used: “deep brain stimulation,” “Parkinson's Disease,” “apathy,” “subthalamic nucleus”, and “globus pallidus.” In addition, we searched the references of identified studies to find other satisfactory articles. This task was completed by two reviewers (S.Z. and S.X.) independently. When disagreements arose, a third reviewer (K.H.) was consulted. The initial study protocol was preregistered at PROSPERO (CRD42022318606).

### 2.2. Inclusion and Exclusion Criteria

The inclusion criteria were as follows: (1) studies were published in English; (2) at least 10 patients recruited in the study; (3) the patients were followed up for at least 3 months; (4) patients were treated with bilateral STN-DBS or bilateral GPi-DBS; (5) presurgery and postsurgery apathy data were obtained through Starkstein Apathy Scale (range from 0 to 42; a score of 14 or greater indicates clinically significant apathy) or Apathy Evaluation Scale [20, 21]; (6) The data were analyzed in the form of the mean (SD).

The exclusion criteria were as follows: (1) studies without original data such as reviews and meta-analyses; (2) duplicate reports with identical data; (3) studies which did not belong to clinical trials; (4) data from nonhuman species; (5) studies included other interventions in addition to DBS; (6) patients with preoperative apathy (defined by Starkstein Apathy Scale or Apathy Evaluation Scale).

### 2.3. Quality Assessment

According to the methodological index for nonrandomized studies (MINORS), two authors (S.Z. and S.X.) independently assessed the quality of each eligible study (Table 1). The MINORS covers 8 different aspects, and each aspect is reported 0–2 (not reported; reported but inadequate; reported and adequate). A score greater than 10 indicates a good quality study [22].

### 2.4. Extraction

The data were extracted from the selected studies by two researchers independently. When disagreements arose, a third researcher was consulted. The extracted data were as follows: first author's name, patients' characteristics, DBS programming, the type of the scale, time of following up and the relevant apathy data in presurgery and postsurgery.

### 2.5. Statistical Analysis

We combined each article using standard meta-analytic methods to estimate the overall efficacy of GPi-DBS and STN-DBS. Revman (Version 5.4, The Cochrane Collaboration, and London, UK) and Statistical Product and Service Solutions (SPSS) were used to analyze available data. The data collected on apathy was evaluated by the Starkstein Apathy Scale and the Apathy Evaluation Scale. The mean difference (MD) and 95% confidence interval (CI) of each outcome were accessed by comparing presurgery and postsurgery stages in both STN-DBS and GPi-DBS. The chi-square and I-square tests were used to measure the statistical heterogeneity between studies. A *P* value <0.05 was considered statistically significant. A random-effects model was used if significant heterogeneity (I^2 > 50%) was found among those studies. Otherwise, a fixed-effect model was used [23]. A sensitivity analysis was performed by excluding each study and reanalyzing the remaining studies. Begg's test was used to assess publication bias. A value of <0.05 for Begg's test was considered statistically significant publication bias [24].

## 3. Results

### 3.1. Characteristics of Eligible Studies

**Figure 1:**
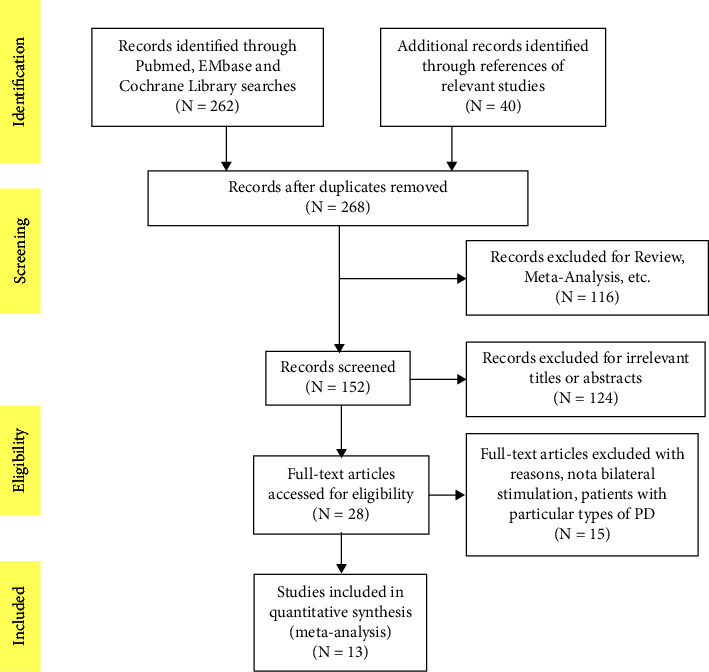
Flowchart of eligible studies.

The flowchart of the study selection process was presented in Figure 1. Overall, 302 studies were initially retrieved and 28 of them were considered eligible. Subsequently, 15 studies were excluded because of their involvement with unilateral stimulation or patients with the particular types of PD. Finally, 13 studies met all the criteria and were included in this meta-analysis.

All the included studies were retrospective studies. The follow-up time ranged from 3 months to 12 months. The sample size was 340, and 230 (67.6%) were accessed using the Apathy Evaluation Scale, the others using the Starkstein Apathy Scale. All patients involved underwent bilateral STN-DBS or bilateral GPi-DBS and were evaluated before and after surgery. The main characteristics were described in Table 2.

### 3.2. Synthesis of the Results of GPi-DBS

**Figure 2:**
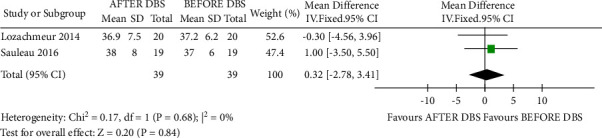
Forest plot for the change in apathy pre-GPi-DBS and post-GPi-DBS.

The heterogeneity between the included studies showed that I^2 = 0%; therefore, the fixed-effects model was used to count the pooled MD. However, based on the comparison of per-operative and postoperative change of apathy score of AES, we found that there was no significant difference in the score between presurgery and postsurgery (MD = 0.32, 95% CI = −2.78–3.41, *P*=0.84, I^2 = 0%) (Figure 2).

### 3.3. Synthesis of the Results of STN-DBS

**Figure 3:**
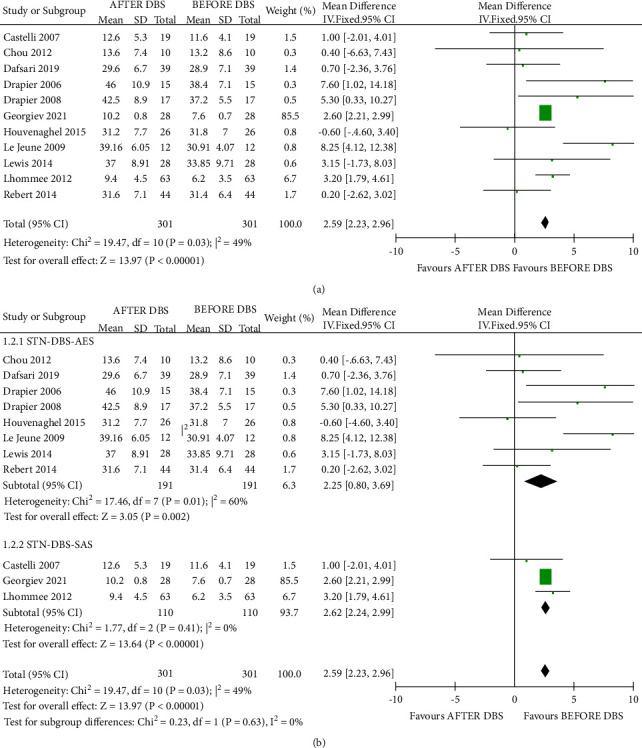
Forest plot for the change in apathy pre-STN-DBS and post-STN-DBS. (a) Overall effects. (b) Subgroup analysis.

The forest plot of the meta-analyses of the STN-DBS studies is shown below (Figure 3). A significantly higher apathy score is found postoperatively than before STN-DBS treatment (MD = 2.59, 95% CI = 2.23–2.96, *P* < 0.00001, I^2 = 49%). Additionally, these two scales were not significantly different (chi^2 = 0.23; *P*=0.63; I^2 = 0%), thus, implying that these two chosen scales did not cause a certain deviation. In the sensitivity analysis, the heterogeneity could be improved by excluding one study with a special design (I^2 = 26%) [27].

### 3.4. Additional Analyses

**Figure 4:**
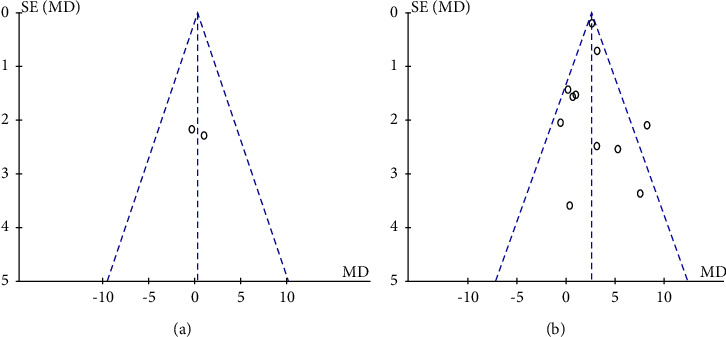
(a) Funnel plot for publication bias in GPi-DBS studies. (b) Funnel plot for publication bias in STN-DBS studies.

In the sensitivity analysis, each study was excluded by turn in order to show the influence of every study contributing to the results. No significant alterations were found in the pooled MD, which showed a high level of stability in our meta-analysis. The Eager's tests provided no evidence for publication bias and there was no small effects bias (Figure 4).

## 4. Discussion

The purpose of this meta-analysis is as follows: (1), to study the difference in apathy before and after GPi-DBS. (2), to update the meta-analysis by including new studies which conclude that apathy is increased in PD patients after STN-DBS. (3), based on results of the meta-analysis, to qualitatively compare the difference in apathy after the stimulations.

We obtained the following results. (1), whether GPi-DBS stimulation reduces or exacerbates apathy in PD patients remains inconclusive. (2) Consistent with the results of the study by Thomas J.C. Zoon et al. [18], apathy scores of PD patients after STN-DBS were significantly increased compared to the preoperative period. (3) Preliminary results suggest that GPi-DBS might affect apathy in PD patients to a lesser extent compared to STN-DBS. As DBS has been clinically adapted as one of the main treatments for patients with advanced PD (APD) [30], these results provide a reference for DBS site selection in APD patients with preoperative apathy and can assist clinicians in developing a reasonable treatment plan, eventually associated directly with the patient's postoperative care.

Current research is focusing on the prognosis of PD patients treated with DBS. The DBS applied to the bilateral STN is the most widely studied and has the largest number of published articles. Previous studies have shown that STN seems to worsen apathy in PD patients [18, 31]. Our study included new observational studies that met the inclusion criteria, and our findings further verified this conclusion. Compared to STN-DBS, fewer studies relate to apathy alterations due to GPi-DBS. The clinical trial carried out by Lozachmeur et al. showed that GPi-DBS does not increase apathy. It can both effectively improve motor symptoms and preserve cognitive function, which is safer than STN-DBS [28]. The results of our study support this conclusion to a certain extent: indeed, the effect of GPi-DBS on postoperative apathy was not significant, but we cannot yet conclude that GPi is safer than STN. The latest research shows that although a small increase in apathy occurs after STN-DBS, other mood-related symptoms such as depression and anxiety have improved to varying degrees [32]. Moreover, there are relatively few studies on the relationships between several negative emotions, such as depression, anxiety, and indifference, etc. In the future, we hope to explore the interaction between several negative emotions and synthetically consider the preoperative status of PD patients to provide a more comprehensive assessment of DBS selection. Many studies have found that greater reductions in levodopa equivalent daily dose (LEDD) are allowed after STN-DBS, compared to GPi-DBS [33–35]. Similar to the former studies, our study showed that the apathy state was deepened after STN-DBS while the difference in apathy was not significant after GPi-DBS, a possible explanation is that there is less reduction of LEDD after GPi-DBS compared to STN-DBS, which leads to lower post-operative apathy scores. In addition, patients on higher doses of levodopa are often offered preferentially STN-DBS as a more advantageous target in many specialized centers. The preference of the target selection may also be part of the potential explanation. Le Jeune et al. demonstrated the correlation between the changes in glucose metabolism and the limbic system, thus, implying STN-DBS may induce apathy directly through limbic system [27]. Mallet and his colleagues suggested that three functional modalities (sensorimotor, cognitive, and emotional) can be combined in the very small volume of the STN [36]. STN can be an integration point of motor, cognitive and emotional components of behavior. Additionally, the dopamine (DA) in the NAcc is involved in motivation, reward, and emotion, recent studies concluded that STN-DBS induced the downregulation of accumbal D_2_R/D_3_R [37]. To summarize, STN-DBS may induce apathy through the limbic system and the downregulation of accumbal DA receptors. The combination of sensorimotor, cognitive, and emotion may also play a role in inducing apathy after STN-DBS.

Our study has some limitations. First, due to the strict inclusion and exclusion criteria, the size of studies included in our analysis was limited. Our study has strict restrictions on the follow-up time; however, Tomas Cartmill et al. found in their meta-regression that the effect of STN-DBS on mood was less affected by age, levodopa dose at follow-up, and stimulation duration [32]. Therefore, as research continues, more credible literature may need to be included in the future to analyze the effects of both stimulation methods on apathy. Second, we did not have access to the raw data, so we failed to include the variables suspected of affecting apathy in the meta-regression to further explore the effect of DBS on apathy. Third, we only explored the effects of STN and GPi-DBS on apathy separately due to the minimal number of randomized clinical trials (RCTs) that investigated the difference in apathy after STN and GPi-DBS simultaneously. In the future, with the inclusion of related RCTs and other studies, we will conduct a meta-analysis of RCTs to make a more accurate quantitative judgment on the difference in the effects of the two stimulation sites on apathy in PD patients.

## 5. Conclusions

In conclusion, apathy is worsened following bilateral STN-DBS. The influence on apathy by GPi-DBS is uncertain. Further studies should focus on the mechanisms of postoperative apathy and the degree of apathy in STN-DBS versus GPi-DBS. It also suggests that a meta-analysis of RCTs is needed to compare the effects of the two stimulation sites on apathy in PD patients.

## Figures and Tables

**Table 1:** MINORS scores of eligible studies (sort by study year and name).

Study	A	B	C	D	E	F	G	H	Total
1 [10]	2	2	2	2	0	2	2	0	12
2 [15]	2	0	2	2	0	2	1	1	10
3 [25]	2	2	2	2	0	2	1	0	11
4 [9]	2	0	2	2	0	2	2	0	10
5 [11]	2	0	2	2	0	2	2	1	11
6 [26]	2	2	2	2	0	2	1	1	12
7 [17]	2	0	2	2	0	2	2	1	11
8 [27]	2	0	2	2	0	2	2	0	10
9 [13]	2	0	2	2	0	2	1	1	10
10 [12]	2	0	2	2	0	2	2	1	11
11 [28]	2	2	2	2	0	2	2	0	12
12 [16]	2	2	2	2	0	2	2	1	13
13 [29]	2	2	2	2	0	2	2	0	12

*Footnote*: DBS programming was all bilateral. NA means not available and NS means not significant. The calculation method of *p* value was as follows: ^a^ means paired-sample Wilcoxon test; ^b^ means Wilcoxon signed-rank test; ^c^ means Friedman test; ^d^ means paired-sample t-test; ^e^ means Mann–Whitney *U* test.

**Table 2:** Studies characteristics.

Study	*N*	Age (y)	M/F	Disease duration	DBS site	Scale	Follow-up	Preoperative score	Postoperative score	*P* value	Preoperative LEDD	Postoperative LEDD	*P* value
Castelli et al. [10]	19	62.1 ± 4.2	8/11	14.7 ± 5.0	STN-DBS	SAS	4 months	11.6 ± 4.1	12.6 ± 5.3	>0.05^d^	1192.5 ± 415.7	571.6 ± 274.8	NA
Chou et al. [15]	17	62.1 ± 6.5	13/4	9.1 ± 5.8	STN-DBS	AES (10)	6 months	13.2 ± 8.6	13.6 ± 7.4	0.96^b^	1164.9 ± 752.9	567.9 ± 512.4	0.001^b^
Dafsari et al. [25]	39	62.8 ± 9.1	25/14	9.6 ± 5.3	STN-DBS	AES	5 months	28.9 ± 7.1	29.6 ± 6.7	0.22^NA^	973.2 ± 484.8	456.1 ± 303.5	<0.001^NA^
Drapier et al. [9]	15	59.7 ± 7.6	10/5	12.2 ± 2.8	STN-DBS	AES	6 months	38.4 ± 7.1	46 ± 10.9	<0.01^a^	1448 ± 400	1127 ± 482	>0.05^a^
Drapier et al. [11]	17	56.9 ± 8.7	11/6	11.8 ± 2.6	STN-DBS	AES	3 months	37.2 ± 5.5	42.5 ± 8.9	0.006^a^	NA	NA	>0.05^a^
Georgiev et al. [26]	28	64.4 ± 6.8	17/11	8.5 ± 4.3	STN-DBS	SAS	12 months	7.6 ± 0.7	10.2 ± 0.8	<0.05^d^	1327.8 ± 117.0	548.0 ± 29.9	<0.05^d^
Houvenaghel et al. [17]	26	56.6 ± 7.4	13/13	11.5 ± 4.5	STN-DBS	AES	3 months	31.8 ± 7.0	31.2 ± 7.7	>0.05^e^	1271.2 ± 555.6	758.0 ± 407.8	NA
Le Jeune et al. [27]	12	57.4 ± 8.0	8/4	11.2 ± 2.4	STN-DBS	AES	3 months	30.91 ± 4.07	39.16 ± 6.05	0.002^a^	1200 ± 426.5	796.66 ± 620	0.02^a^
Lewis et al. [13]	28	61.1 ± 8.9	17/11	12.4 ± 6.7	STN-DBS	AES	12 months	33.85 ± 9.71	37.00 ± 8.91	0.023^NA^	832 ± 426	359.2 ± 264.5	<0.001^NA^
Lhommée et al. [12]	63	57.8 ± 7.2	40/23	10.5 ± 3.1	STN-DBS	SAS	12 months	6.2 ± 3.5	9.4 ± 4.5	<0.001^a^	1026 ± 459	284 ± 312	<0.001^a^
Lozachmeur et al. [28]	20	60.1 ± 9.1	10/10	13.3 ± 5.4	GPi-DBS	AES	3 months	37.2 ± 6.2	36.9 ± 7.5	0.36^C^	1348.7 ± 510.2	1368.3 ± 428.2	0.94^C^
Robert et al. [16]	44	56.3 ± 7.5	24/20	11.4 ± 4.1	STN-DBS	AES	3 months	31.4 ± 6.4	31.6 ± 7.1	NA	1280.8 ± 632.4	889.9 ± 209.3	NA
Sauleau et al. [29]	19	61 ± 8	9/10	NA	GPi-DBS	AES	4 months	37 ± 6	38 ± 8	>0.05^NA^	1415 ± 587	1372 ± 434	>0.05^NA^

DBS programming was all bilateral. NA means not available and NS means not significant. The calculation method of *p* value was as follows: a means paired-sample Wilcoxon test, b means Wilcoxon signed-rank test, c means Friedman test, d means paired-sample *t*-test, e means Mann-Whitney U test.

## Data Availability

The datasets used or analyzed to support the findings of this study are available from the corresponding author on reasonable request.
